# Dawn- and dusk-phased circadian transcription rhythms coordinate anabolic and catabolic functions in *Neurospora*

**DOI:** 10.1186/s12915-015-0126-4

**Published:** 2015-02-24

**Authors:** Cigdem Sancar, Gencer Sancar, Nati Ha, François Cesbron, Michael Brunner

**Affiliations:** Heidelberg University Biochemistry Center, Heidelberg, Germany; University of Heidelberg Biochemistry Center, Im Neuenheimer Feld 328, Heidelberg, D-69120 Germany

**Keywords:** Circadian, WCC, CSP1, Transcriptome, RNA-seq, ChIP-seq, RNAPII, Metabolism

## Abstract

**Background:**

Circadian clocks control rhythmic expression of a large number of genes in coordination with the 24 hour day-night cycle. The mechanisms generating circadian rhythms, their amplitude and circadian phase are dependent on a transcriptional network of immense complexity. Moreover, the contribution of post-transcriptional mechanisms in generating rhythms in RNA abundance is not known.

**Results:**

Here, we analyzed the clock-controlled transcriptome of *Neurospora crassa* together with temporal profiles of elongating RNA polymerase II. Our data indicate that transcription contributes to the rhythmic expression of the vast majority of clock-controlled genes (ccgs) in *Neurospora*. The ccgs accumulate in two main clusters with peak transcription and expression levels either at dawn or dusk. Dawn-phased genes are predominantly involved in catabolic and dusk-phased genes in anabolic processes, indicating a clock-controlled temporal separation of the physiology of *Neurospora*. Genes whose expression is strongly dependent on the core circadian activator WCC fall mainly into the dawn-phased cluster while rhythmic genes regulated by the glucose-dependent repressor CSP1 fall predominantly into the dusk-phased cluster. Surprisingly, the number of rhythmic transcripts increases about twofold in the absence of CSP1, indicating that rhythmic expression of many genes is attenuated by the activity of CSP1.

**Conclusions:**

The data indicate that the vast majority of transcript rhythms in *Neurospora* are generated by dawn and dusk specific transcription. Our observations suggest a substantial plasticity of the circadian transcriptome with respect to the number of rhythmic genes as well as amplitude and phase of the expression rhythms and emphasize a major role of the circadian clock in the temporal organization of metabolism and physiology*.*

**Electronic supplementary material:**

The online version of this article (doi:10.1186/s12915-015-0126-4) contains supplementary material, which is available to authorized users.

## Background

Circadian clocks are molecular oscillators that coordinate metabolism, physiology and behavior of organisms with daily environmental changes [1-3]. In eukaryotes, the robustness of circadian oscillations is dependent on cell-autonomous interconnected transcriptional-translational feedback loops. These circadian oscillators drive rhythmic expression of clock-controlled genes (ccgs) in various organisms [4-7]. In mammals, the circadian clock coordinates metabolic pathways, such as glycolysis, gluconeogenesis, fatty acid oxidation and xenobiotic detoxification [8-11]. Disruption of the circadian oscillator in mammals is associated with metabolic pathologies, premature aging and cancer [12-14]. In plants, misalignment of the circadian clock with the external light–dark cycle results in lower chlorophyll production and slower growth [15,16]. The mechanisms generating circadian expression rhythms and circadian phase are complex. It is therefore important to identify in a comprehensive manner the genes that are controlled by the circadian clock and understand the molecular mechanisms underlying rhythmic gene expression. Gene expression analyses in a variety of organisms suggested that 2% to 15% of their transcriptomes are expressed in a circadian fashion with different phases throughout the day [2,17-19]. Circadian chromatin modifications and transcribing RNA polymerase II (RNAPII) profiles indicate a crucial role of circadian transcription in the orchestration of rhythmic gene expression [6,20]. Moreover, recent genome-wide studies in animals suggest that post-transcriptional processes contribute substantially to the generation of rhythmic transcript levels in addition to rhythmic transcription [4-6,21].

The white collar complex (WCC) is the core transcription activator of the circadian oscillator of *Neurospora crassa* [22,23]. It is composed of two GATA type transcription factors, white collar-1 (WC1) and white collar-2 (WC2) [24,25]. WC1 is the main blue-light photo-receptor of *Neurospora* [26,27]*.* WCC is activated by light and required for the synchronization of the circadian clock with exogenous light–dark cycles [24,27]. Most ccgs identified previously had maximum expression levels around dawn, that is, at a time when the WCC is highly active [28-30] and also at dusk [31]. We, in collaboration with colleagues, recently presented evidence that the WCC controls expression of about 24 transcription factors [32], which have the potential to transduce circadian information to downstream genes. Such second tier circadian transcription factors may generate different phases of circadian gene expression. In particular, the transcription repressor CSP1, which is rhythmically expressed with morning-specific peaks in abundance and repressing activity, has the potential to modulate rhythmic expression of several target genes with an evening-specific phase [33,34].

Here, we have compared rhythmic transcript abundance and transcription profiles of transcribing RNAPII to determine the circadian transcriptome of *Neurospora* and assess the contribution of transcription versus post-transcriptional processes to gene expression rhythms controlled by the circadian clock. By frequent sampling we have obtained detailed phase information. Moreover, we analyzed the roles of WCC and CSP1 on circadian gene expression rhythms.

## Results

### Transcription-based rhythmic gene expression in two circadian phases

**Figure 1:**
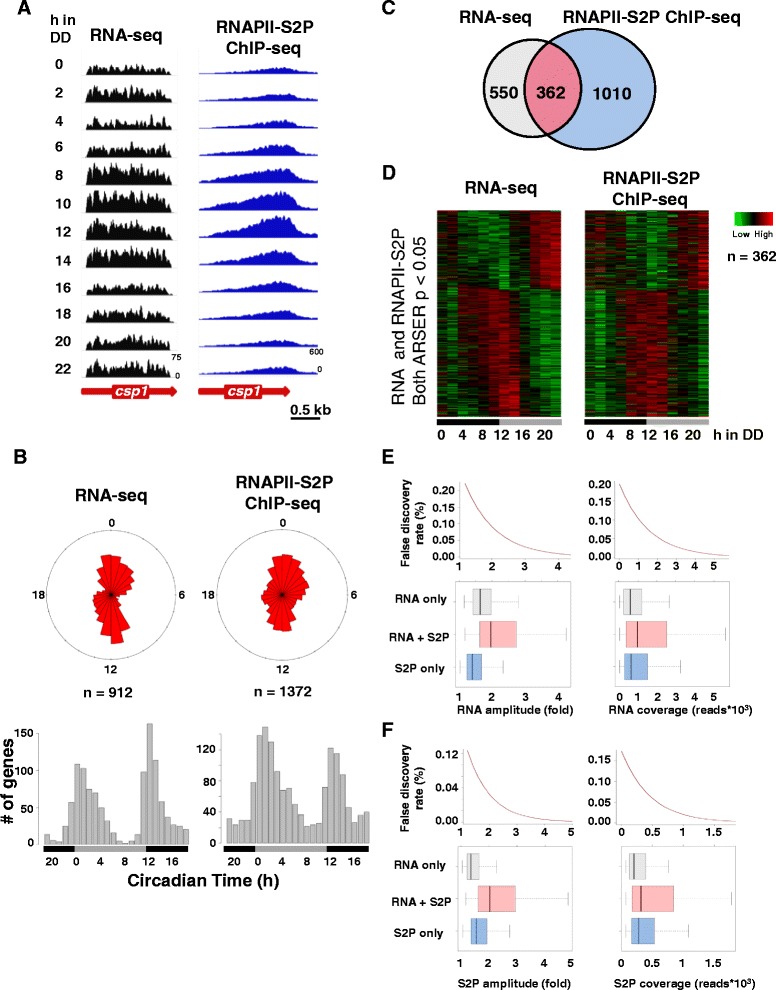
**Circadian gene expression rhythms in** ***Neurospora.*** **(A)** Circadian time course of RNA-seq (left panel) and RNAPII-S2P ChIP-seq (right panel) reads of *csp1* in constant dark. RNAPII-S2P signals were generally enriched at the end of the genes, indicating that the phospho-S2 antibody was specific for the elongating/terminating polymerase [37,38]. **(B)** Rose plot (upper panel) and histogram (lower panel) of the phase distribution of rhythmic RNA levels and RNAPII-S2P profiles by ARSER. **(C)** Venn diagram showing the overlap of genes with rhythmic RNA levels and RNAPII-S2P profiles. **(D)** Heat-maps showing the relative RNA levels (left panel) and RNAPII-S2P occupancy (right panel) of genes with robust rhythms identified by ARSER (*P* value <0.05 for both RNA and RNAPII-S2P). **(E)** Box-plots showing the RNA amplitude (left panel) and coverage (right panel) of the rhythmic genes identified only by RNA-seq (RNA only, grey), identified only by RNAPII-S2P ChIP-seq (S2P only, blue) and identified by both methods (RNA + S2P, light red). False discovery rates (FDR %) versus amplitude and coverage are shown in the panels above the corresponding box-plots. **(F)** Same as in **E**, shown for RNAPII-S2P amplitude (left panel) and coverage (right panel). CHIP-seq, chromatin immunopreciptation sequencing; RNAPII, RNA polymerase II.

To identify circadian rhythms in gene expression in a genome-wide manner*, Neurospora* cultures were entrained to 11/11 hour light/dark cycles for two days and then released into constant darkness. Samples were harvested in two-hour intervals in constant darkness over a time period of 22 hours, which corresponds to the endogenous free-running period of the circadian clock of *Neurospora* [35]. RNA levels were quantified by next-generation sequencing (RNA-seq) (see Additional file 1: Table S1). To identify rhythmically transcribed genes we analyzed the circadian profiles of elongating RNAPII by chromatin immunoprecipitation-sequencing (ChIP-seq) with an antibody recognizing serine-2 phosphorlyated C-terminal domain repeats of the large subunit of RNAPII (RNAPII-S2P) from an independent time-course experiment (see Additional file 1: Table S1). Examples of circadian RNA abundance rhythms and the corresponding RNAPII-S2P occupancy profiles of previously identified clock-controlled genes are shown in Figure 1A and Additional file 2: Figure S1A. Using ARSER [36] we identified 912 genes with cycling RNA levels and 1,372 genes with rhythmic RNAPII-S2P occupancy (*P* <0.05) (Figure 1B) (see Additional file 3: Table S2). Both rhythmic transcript levels and transcribing RNAPII profiles cluster into two main phases, with peak levels either late night to early morning (dawn-phased) or late day to early evening (dusk-phased) (Figure 1B). A total of 362 genes displayed significant rhythms in both transcript levels as well as RNAPII-S2P occupancy (Figure 1C) (see Additional file 4: Table S3). The transcribing RNAPII profiles and the corresponding transcript abundance rhythms of these genes were in phase (Figure 1D), suggesting that the expression rhythms were generated on the level of transcription. The amplitudes of the RNA abundance and transcribing RNAPII rhythms were generally rather low (Figure 1E and F). We suspected that the rather low overlap of RNA abundance and RNAPII-S2P occupancy rhythms (approximately 40%) may be due to the failure reliably to detect low amplitude rhythms and genes with low expression levels. In accordance, genes with significant RNA abundance and transcription rhythms had a higher coverage and amplitude in both RNA-seq and RNAPII-S2P ChIP-seq (Figure 1E and F). Based on randomly shuffled data, we estimated that the false discovery rate (FDR) increased substantially with decreasing coverage and amplitude (Figure 1E and F). Highly expressed genes with high amplitude (low FDRs) should be highly confident clock-controlled genes. In fact, analysis of 87 robustly oscillating transcripts (>3 fold amplitude, FDR <0.05) revealed that 67 (approximately 80%) displayed in-phase RNAPII-S2 transcription rhythms (see Additional file 4: Table S3). For 10 of the 87 genes, a transcription rhythm was probably not detected due to an outlier and the remaining 10 genes had a low RNAPII-S2P coverage (median <200 reads). Together the data suggest that highly rhythmic genes display in-phase transcription and RNA abundance rhythms.

**Figure 2:**
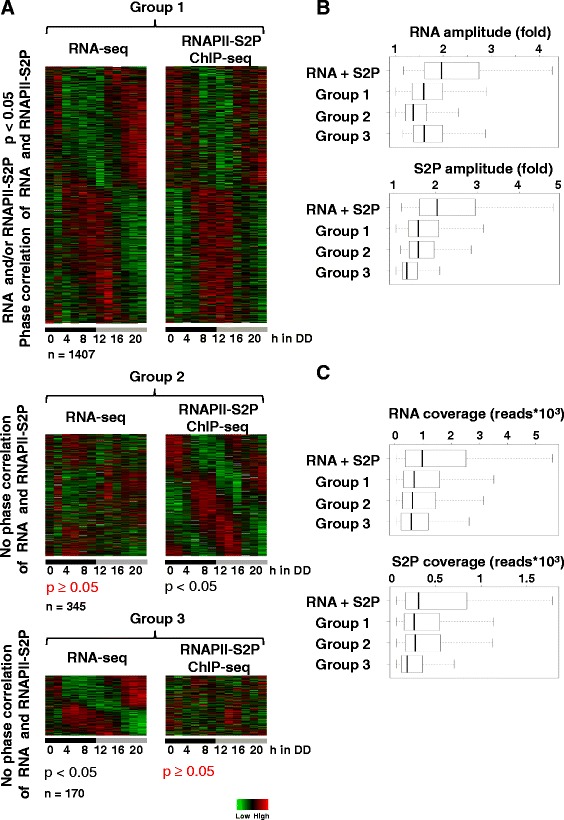
**The majority of the clock-controlled genes have similar RNA and RNAPII-S2P profiles. (A)** Heat-maps showing the relative RNA levels (left panels) and RNAPII-S2P occupancy profiles (right panels) for rhythmically expressed genes. Group 1: genes with similar phases of RNA levels and RNAPII-S2P. One or both profiles were identified as rhythmic by ARSER (*P* <0.05). Group 2: genes with significantly rhythmic RNAPII-S2P profiles and non-significantly rhythmic, out of phase RNA abundance profile. Group 3: genes with significantly rhythmic RNA profiles and non-significantly rhythmic, out of phase RNAPII-S2P occupancy profile. **(B)** Amplitude of RNA-seq (upper panel) and RNAPII-S2P ChIP-seq profiles (lower panel) of the three groups in comparison to 362 genes with both significant RNA and RNAPII-S2P rhythms (RNA + S2P). **(C)** Same as in B, shown for RNA (upper panel) and RNAPII-S2P ChIP-seq coverage (lower panel). CHIP-seq, chromatin immunopreciptation sequencing; RNAPII, RNA polymerase II.

In order to detect the group of potentially rhythmically transcribed genes, we analyzed all candidates with a significant RNA abundance rhythm (*P* <0.05) that had in addition a non-significant RNAPII-S2P rhythm (*P* ≥0.05) in phase with the transcript rhythm (see Additional file 2: Figure S1B) and vice versa all genes with a significant RNAPII-S2P rhythm (*P* <0.05) and a non-significant RNA abundance rhythm in the same phase (see Additional file 2: Figure S1C). An additional 1,045 genes met these criteria. Combined analyses of these 1,045 genes together with the 362 genes with significant RNA abundance and RNAPII-S2P occupancy rhythms (Figure 2A) suggest that, in total, about 1,407 genes were rhythmically transcribed under the growth conditions used (Group 1) (see Additional file 4: Table S3).

A total of 345 rhythmically transcribed genes (RNAPII-S2P rhythm, *P* <0.05) did not show corresponding in phase transcript abundance rhythms (Group 2) (see Additional file 5: Table S4). Heat-map analysis suggested that the transcript levels of these genes increase with a substantial delay after the transcription rhythm (RNAPII-S2P profile). Hence, these RNAs could have a long half-life resulting in a delayed phase and a blunted abundance rhythm [7]. Moreover, RNAPII-S2P amplitude of the oscillations of these genes was lower compared to genes identified by both methods (Figure 2B).

Finally, 170 genes with significant RNA abundance rhythms did not exhibit rhythmic RNAPII-S2P occupancy profiles (Group 3) (see Additional file 6: Table S5). This class of genes is potentially interesting since the RNA abundance rhythms might be based on hitherto unknown post-transcriptional mechanisms that are controlled by the circadian clock. However, the average RNAPII-S2P read coverage of these genes was low in comparison to genes that have significant profiles in RNAPII-S2P occupancy and RNA abundance (Figure 2C) (*P* <10^−4^). Thus, we cannot rigorously exclude transcription based expression rhythms of these genes. Together, the data suggest that expression of ccgs in *Neurospora* is predominantly associated with rhythmic transcription. Transcription independent generation of circadian transcript abundance rhythms, for example, on the level of rhythmic RNA turnover, may thus not be a major pathway used by the circadian clock.

A very recent RNA-seq analysis of three replicate time-courses by Hurley *et al*. [31] identified 872 genes with circadian RNA abundances rhythms. Of these 872 genes, 697 were expressed under our growth conditions and 327 of them were assigned as rhythmic in our study (see Additional file 7: Figure S2A, Additional file 4: Table S3). The remaining 370 genes were expressed at low levels under our conditions and oscillated with low amplitude in our and the Hurley *et al*. study (see Additional file 7: Figure S2B-D). A comparison of RNA abundance rhythms and RNAPII-S2P profiles of the rhythmic genes identified by both studies suggests that these genes are controlled on the level of transcription (see Additional file 7: Figure S2E).

Hurley *et al*. found among 187 luciferase reporters two genes (*ccg1* and *ccg9*) with rhythmic RNA but no luciferase rhythm of the corresponding reporter gene (compare Additional file 8: Figure S6 and Additional file 9: Table S10 in Hurley *et al*.), suggesting a posttranscriptional regulation. We analyzed the RNAPII-S2P profiles of these genes by ChIP-seq and ChIP-PCR and found that both genes are rhythmically transcribed (see Additional file 7: Figure S2F and G).

### WCC and CSP1 are major determinants of circadian phase

**Figure 3:**
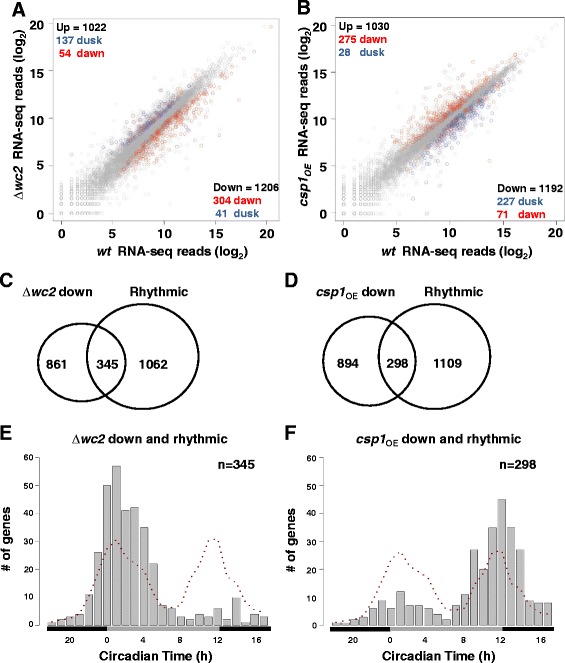
**WCC and CSP1 are determinants of dawn- and dusk-specific gene expression.** Dawn-phased genes are preferentially downregulated in ∆*wc2* and upregulated in *csp1*_*OE*_ while dusk-phased genes are upregulated in ∆*wc2* and downregulated in *csp1*_*OE*_. RNA-seq reads in log_2_ scale **(A)** *wt* versus ∆*wc2* strain and **(B)** *wt* versus *csp1*_*OE*_ strain. For comparability of gene expression levels of ∆*wc2* (arrhythmic) with *wt* and *csp1*_*OE*_ all strains were grown in constant light to mask circadian expression rhythms. The numbers of genes that are significantly up- and downregulated in ∆*wc2* and *csp1*_*OE*_ are indicated (black numbers). Genes that are rhythmically expressed in darkness in *wt* (Figure 2A, n = 1,407) and significantly misregulated in ∆*wc2* or *csp1*_*OE*_ are represented by red circles and numbers for dawn-phased genes and by blue circles and numbers for dusk-phased genes. In total, 1,137 of 1,407 rhythmically expressed genes are misregulated in ∆*wc2* and/or *csp1*_*OE*_. **(C and D)** Venn-diagram showing the overlap between rhythmically expressed genes in *wt* (n = 1,407) and the genes with lower expression in **(C)** ∆*wc2* and **(D)** *csp1*_*OE*_. **(E and F)** Histogram plot showing the phase distribution of **(E)** 345 WCC regulated and rhythmic genes and (F) 289 CSP1 regulated and rhythmic genes. The dotted lines correspond to the expected phase distribution of the genes based on the all rhythmic genes (n = 1,407). RNA-seq data for CSP1 regulated genes are from a previously published study [32].

The morning-specific core circadian transcription factor WCC is rhythmically inactivated by clock-dependent phosphorylation [39,40]. When the function of WCC is compromised light- or clock-dependent transcription is essentially abolished [22,41,42]. To identify genes that are potentially regulated by WCC we analyzed the transcriptomes of light-grown cultures of *wt* and ∆*wc2* strains by RNA-seq. The expression of 1,206 genes was reduced in ∆*wc2* and 345 of these genes were transcribed in rhythmic fashion in *wt* in constant darkness (Figure 3A and C) (see Additional file 10: Table S6)*.* We have previously shown that WCC controls morning specific expression of the transcription repressor CSP1 [33,34]. CSP1 is a global glucose-dependent regulator with the potential to repress 1,192 genes (Figure 3B). A total of 298 putative target genes of CSP1 were transcribed in circadian fashion (Figure 3D) (see Additional file 11: Table S7)*.* The expression rhythms of the majority of the WCC-dependent ccgs were dawn-phased while most CSP1-repressed rhythmic genes were dusk-phased in accordance with the morning specific activating and repressing activity, respectively, of these transcription factors (Figure 3E and F).

We then analyzed the expression phases of ccgs that might be indirectly induced by the CSP1 repressor (303 genes) or indirectly repressed by the WCC activator (191 genes) (see Additional file 12: Figure S3). These ccgs were also expressed in either a morning- or evening-specific manner. The 191 ccgs that were upregulated in ∆*wc2* were preferentially dusk-phased (Figure 3A and Additional file 12: Figure S3A) (see Additional file 10: Table S6) while the 303 ccgs upregulated in a CSP1 overexpressing strain (*csp1*_*OE*_) were mainly dawn-phased (Figure 3B and Additional file 12: Figure S3B) (see Additional file 11: Table S7). Together, WCC and CSP1 appear to regulate, directly or indirectly, at least 1,137 of the 1,407 genes (approximately 80%) that were rhythmically transcribed in *wt*. Indeed, we found that 457 of 1,407 rhythmically expressed genes have CSP1 binding sites in their upstream regions suggesting a direct regulation by CSP1 (Additional file 11: Table S7). Hence, WCC and CSP1 are major determinants of clock-controlled transcription and circadian phase.

**Figure 4:**
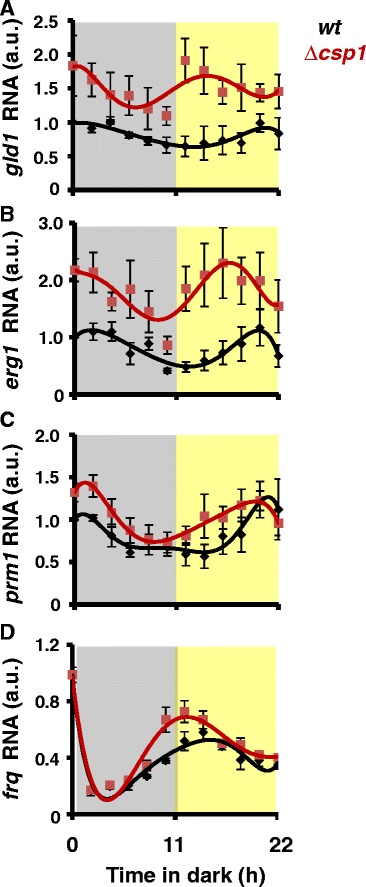
**CSP1 shifts expression phases towards subjective dusk.** Circadian expression profiles determined by qRT-PCR of **(A)** *gld1* (*ncu04923*)*,* **(B)** *erg1 (ncu04156)*, **(C)** *prm1 (ncu07459)* and **(D)** *frq (ncu02265)* RNA in *wt* (black symbols and trend-lines) and ∆*csp1* (red symbols and trend-lines). RNA levels of *wt* prior to the light–dark transfer of cultures (t = 0 hours) were normalized to 1 within each independent experiment. ± SEM, n = 3. Dark and light shaded areas indicate subjective night and subjective day, respectively.

In order to further investigate the role of CSP1 in circadian gene regulation, we analyzed the expression of several CSP1-repressed genes in *wt* and ∆*csp1* by RT-qPCR (Figure 4A-C)*.* In the absence of CSP1 the levels of *glycerol dehydrogenase 1 (gld1*) RNA were elevated and the expression phase was shifted from evening to the early day (Figure 4A). This major phase change emphasizes the dominant role of CSP1 on the evening specific expression of *gld1.* The expression levels of *erg-1* (encoding a sterol isomerase) and to a lesser extent of *prm-1* (encoding a protein arginine N-methyltransferase) were also elevated in ∆*csp1* and the expression phases were shifted towards morning (Figure 4B and C). Interestingly, the CSP1 target genes analyzed above were rhythmically expressed in ∆*csp1*, suggesting that rhythmic transcription activators control their expression in addition to the rhythmic CSP1 repressor. The phase shifts towards subjective morning in ∆*csp1* suggests rather that these evening-specific genes are controlled by morning-specific circadian transcription activators.

We have previously shown that CSP1 inhibits *wc1* transcription and thereby regulates WCC expression levels [34]. Therefore, we also analyzed the expression of *frq*, which is a direct target of WCC. In ∆*csp1* the levels of *frq* RNA were slightly elevated and the expression phase was slightly advanced (Figure 4D), consistent with the shorter period rhythm observed in ∆*csp1* in high glucose conditions [34].

**Figure 5:**
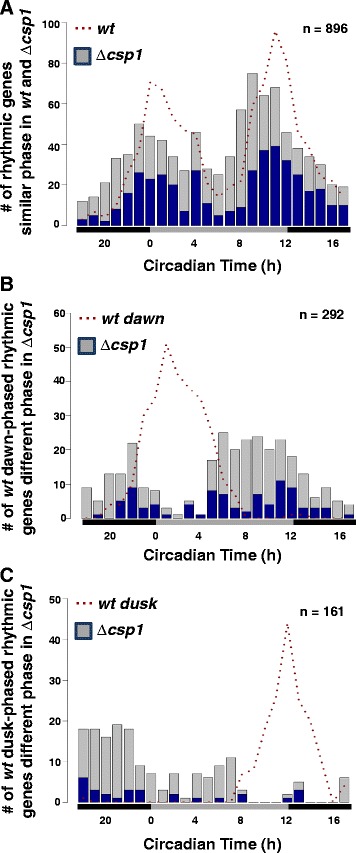
**Phase analysis of** ***wt*** **rhythmic genes in** **∆** ***csp1.*** **(A)** Bar-plots showing the phase distribution of genes that are rhythmic in *wt* and have a similar phase in ∆*csp1.* Phase distribution of **(B)** *wt* dawn-phased genes that have a different phase in ∆*csp1* and **(C)** *wt* dusk-phased genes that have a different phase in ∆*csp1*. Dotted lines represent the phase distribution of rhythmic genes in *wt*. Blue and grey colors correspond to the fraction of genes that have significant (blue) (*P* <0.05) and non-significant (grey) (*P* ≥0.05) RNA rhythms in ∆*csp1* as identified by ARSER.

In order to assess the contribution of CSP1 on the generation of rhythmic transcripts, we determined the circadian transcriptome of ∆*csp1* by RNA-seq (see Additional file 13: Table S8). At first, we analyzed the phases of the 1,407 genes that were rhythmically transcribed in *wt.* A total of 1,349 of these genes were expressed in ∆*csp1*. Of these 1,349 expressed genes, 896 (approximately 66%) showed similar circadian expression patterns in *wt* and ∆*csp1* (Figure 5A and Additional file 14: Figure S4A) whereas 453 ccgs (approximately 34%) were expressed in different phases in ∆*csp1.* A total of 292 ccgs with dawn-specific expression in *wt* shifted to predominantly dusk-phased expression in ∆*csp1* (Figure 5B) and 161 ccgs that showed dusk-phased rhythms in *wt* shifted to predominantly late-night-specific expression in ∆*csp1* (Figure 5C)*.* The data suggest that CSP1 affects the expression phase of morning- and evening-specific ccgs.

Interestingly, we identified 1,867 genes that were rhythmically expressed in ∆*csp1* (*P* <0.05) but not in *wt* (see Additional file 14: Figure S4B and Additional file 15: Table S9). The expression rhythms of these ∆*csp1*-specific ccgs also were clustered in two phases. About 2/3 of the ∆*csp1*-specific ccgs were expressed with a dusk- and 1/3 with a dawn-specific phase. Intriguingly, the dusk- and dawn-phased ∆*csp1*-specific ccgs also clustered in two groups in *wt*, displaying rather complex temporal expression profiles (see Additional file 14: Figure S4B). The data suggest that expression of these genes is clock-controlled. However, under the conditions analyzed, that is, 2% glucose-containing medium, their circadian regulation in *wt* appears to be blunted by the glucose-dependent activity of CSP1. Examples of these ∆*csp1*-specific genes are shown in Additional file 14: Figure S4C.

Together, the data suggest that CSP1 regulates the phase and amplitude of both dusk-phased and dawn-phased rhythmic genes. The over-expression of CSP1 indicates that dusk-phased genes are repressed by CSP1 in the subjective morning whereas dawn-phased genes are indirectly activated via unknown pathways. Rhythmic expression of only a fraction of dusk-phased genes is affected in a ∆*csp1* strain, suggesting that other transcription factors (TFs) contribute to evening-specific gene expression. The increased accumulation of WCC in ∆*csp1* [34] could additionally affect expression of dawn- and dusk-phased ccgs, suggesting a further mechanism by which CSP1 may affect circadian gene expression.

### Temporal separation of biological functions by the circadian clock

**Figure 6:**
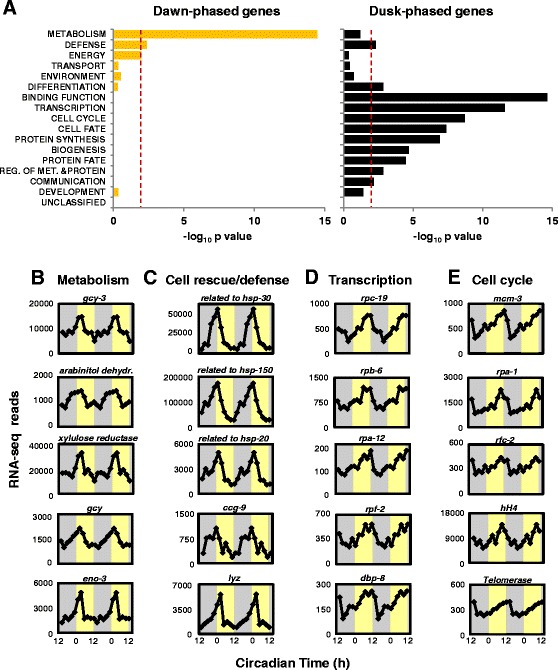
**Distinct functional bias of dawn- and dusk-phased genes. (A)** The enrichment of the functional categories of dawn- and dusk-phased genes is shown. In total, 1,407 genes with in-phase RNA and RNAPII-S2P profiles together with 170 genes with rhythmic RNA levels but not rhythmic RNAPII-S2P occupancy were analyzed with MIPS FunCat [44] using ‘*Neurospora crassa*’ database. The dotted red line shows the border for significance *P* <0.01. RNA expression profiles of selected genes involved in **(B)** metabolism, **(C)** cell rescue and defense, **(D)** transcription, and **(E)** cell cycle are shown. RNA-seq reads were double plotted. Light- and dark-shaded areas correspond to subjective day (CT 0 to 12) and night (CT 12 to 0), respectively. The expression levels (Y-axis) are shown as mapped normalized reads. FunCat, Functional Catalogue; MIPS, Munich Information Center for Protein Sequences. RNAPII, RNA polymerase II.

An analysis of the functional categories of genes controlled by the circadian clock of *Neurospora* revealed an astonishingly clear functional separation between dawn- and dusk-phased genes. Genes with functions related to metabolism and cell rescue/defense were enriched in the group of dawn-phased ccgs, whereas gene categories related to growth, such as cell cycle, protein synthesis and biogenesis of cellular components, were enriched among the dusk-phased genes (Figure 6A) (see Additional file 9: Table S10). Examples of the dawn-phased metabolic genes involved in the utilization of carbon sources are shown in Figure 6B. The genes *gcy-3, L-arabinitol 4-dehydrogenase* and *L-xylulose reductase*, which are required for the utilization of L-arabinose, and *gcy*, a putative D-xylose reductase gene (see Additional file 16: Figure S5A), were expressed with a dawn-phased rhythm. L-arabinose and D-xylose are the major pentoses of plant hemicellulose and pectin [43], that is, of natural substrates of *Neurospora.* L-arabinose and D-xylose are converted to xylitol, which is further processed via the pentose phosphate pathway and, eventually, funneled into glycolysis. Interestingly, *eno-3,* encoding for the glycolytic enolase, also displayed a robust dawn-phased expression rhythm.

Examples of dawn-phased genes involved in cell rescue and defense are shown in Figure 6C. The dawn-phased expression of heat-shock factors may contribute to the adaptation of *Neurospora* to higher temperature expected during the day. In order to prevent desiccation during the day fungi (as well as bacteria and plants) synthesize trehalose. *Neurospora* can synthesize trehalose via a one-step pathway by the trehalose synthase encoded by the morning-specific *clock-controlled gene-9* (*ccg-9*) [45] and via a two-step reaction requiring α, α-trehalose phosphate synthase and trehalose phosphatase (see Additional file 16: Figure S5B). The corresponding genes are rhythmically expressed with a morning-specific phase (Figure 6C, Additional file 1: Table S1 and Additional file 3: Table S2). It is also noteworthy that the *Neurospora lysozyme* gene (*lyz*) was rhythmically expressed with peak levels at dawn (Figure 6C). Although the function of lysozymes in fungi is not well characterized, a potential anti-microbial activity might preferentially be required during the day, when bacterial growth is supported by elevated temperature.

A recent study revealed a role of the circadian clock in the coordination of ribosome biogenesis in mammals [46]. In *Neurospora*, the genes *rpc-19, rpb-6* and *rpa-12*, which encode subunits of RNA polymerase I, were rhythmically expressed with a dusk-phased peak (Figure 6D). Moreover, *rpf-2* and *dbp-8,* genes involved in rRNA maturation and ribosome assembly, were rhythmically expressed in an evening-specific manner. The data suggest that the circadian clock of *Neurospora* might affect ribosome biogenesis and protein translation.

Genes involved in DNA replication and cell division were also enriched among the dusk-phased circadian genes (Figure 6E). For example, *mcm-3, mcm4* and *mcm-5* encoding subunits of DNA replication licensing factor, required to initiate DNA replication in eukaryotes [47], were rhythmically expressed with a peak around dusk. The other subunits, *mcm-2, 6,* and *7*, did not qualify as rhythmic genes by our criteria but appeared to be expressed with low-amplitude dusk-phased rhythms (see Additional file 8: Figure S6A). In addition *rpa-1* and *rpa-2*, encoding the subunits of hetero-trimeric replication protein A, also showed circadian expression with a similar peak time with *mcm* genes. (Figure 6E and Additional file 8: Figure S6B). Furthermore, *rfc-2*, which encodes a subunit of the hetero-pentameric clamp loader complex, was expressed with a dusk-phased rhythm. The genes encoding the remaining subunits of the clamp loader were potentially also expressed in an evening specific manner (see Additional file 8: Figure S6C). Moreover, the genes encoding the core histones hH2A, hH2B, hH3 and hH4 were expressed at higher levels during dusk (Figure 6E, Additional file 8: Figure S6D). Finally, the gene encoding for the reverse transcriptase subunit of telomerase was robustly rhythmic with an evening specific phase (Figure 6E). The data strongly suggest that genes required for various aspects of cell division and growth are expressed in an evening-specific manner under the control of the circadian clock of *Neurospora.*

Together, the bi-phasic clustering of ccgs and the pronounced separation of the corresponding gene functions suggest a global and comprehensive temporal coordination of gene expression by the circadian clock to support rhythmic growth of *Neurospora.*

**Figure 7:**
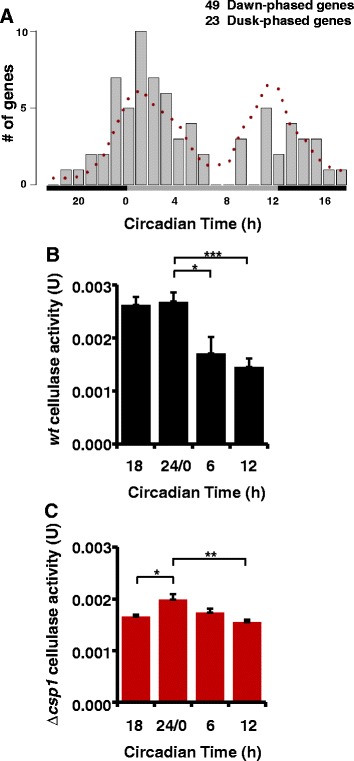
**Circadian changes of cellulase activity. (A)** Phase distribution of rhythmically expressed genes that were previously described to be upregulated upon growth of *Neurospora* on ground plant material or cellulose [48]. The dotted line shows the expected phase distribution of the 72 genes on the basis of the phases of all rhythmic genes. Cellulase activity of **(B)** *wt* and **(C)** ∆*csp1* at different times of the day (± SEM, four to six experiments). *P <0.05, **P <0.01, ***P <0.001.

In nature *Neurospora* is often detected on scorched or decaying plant material. Recently, 304 genes were found to be upregulated when *Neurospora* was grown in liquid medium with ground plant tissue (miscanthus) or crystalline cellulose (Avicel) as carbon sources [48]. Under the conditions used in our study, that is, glucose containing medium, 72 of these genes were under circadian control with predominantly dawn-phased rhythms (Figure 7A and Additional file 17: Figure S7A), including genes required for the mobilization and utilization of cellulose. In order to assess the physiological relevance of circadian expression of these genes, *Neurospora* was grown on Avicel-containing medium and cellulase activity associated with mycelial pads was measured in a time-of-day dependent manner in *wt* and ∆*csp1.* Cellulase activity associated with *wt* mycelia was higher at late night to early morning (Figure 7B) coinciding with the expression rhythm of cellulose utilization enzymes. In ∆*csp1* the dawn-phased cellulase activity was reduced, resulting in a blunted activity rhythm (Figure 7C).

Our data suggest that clusters of functionally related genes are expressed in a morning- or evening-specific manner under the control of the *Neurospora* clock.

## Discussion

In this study we analyzed circadian gene expression in *Neurospora* on a genome-wide level to reveal organizational principles of clock-regulation. Temporal profiles of the circadian transcriptome and elongating RNAPII revealed 912 and 1,372 genes with significant rhythms, respectively. The two hour experimental sampling frequency (12 time points) provided rather reliable amplitude and phase information even from a single replicate. A ‘false discovery’ estimation based on randomly shuffled data indicated that the confidence for detecting clock-controlled genes increases with sequence coverage and amplitude of the RNAPII-S2P ChIP-seq and RNA-seq. Based on the false discovery estimation many sequencing replicates would be required to reliably identify all lowly expressed clock-controlled genes. However, assuming that such genes are rhythmically regulated in similar fashion as highly expressed genes, a complete list of such genes *per se* may not be of interest.

We show that the RNA abundance and transcription rhythms clustered mainly in two phases with peak levels around dawn and dusk. At least 1,407 genes appear to be transcribed in circadian fashion by our criteria (same phase of RNAPII and RNA abundance rhythms and at least one rhythm with a *P*-value <*0*.05). Another 345 genes are rhythmically transcribed but may encode rather stable transcripts that accumulate with a delayed phase and blunted amplitude. The abundance levels of 170 transcripts cycled in circadian fashion without an apparent rhythm of transcribing RNAPII. Rhythmic expression of these genes could potentially be regulated by post-transcriptional clock-controlled mechanisms. However, these genes showed very low RNAPII occupancy and low RNA expression levels so that the absence of an apparent transcription rhythm might, in many cases, be due to detection problems. Hence, the vast majority of rhythmically expressed genes of *Neurospora* appear to be controlled on the level of rhythmic transcription. Clock-dependent posttranscriptional mechanisms could in principle synergistically contribute to cycling RNA abundance levels to support rhythmic transcription. However, circadian expression of only a very small fraction of rhythmic genes might be exclusively controlled on a post-transcriptional level.

A recent analysis of clock-regulated genes in *Neurospora* suggested post-transcriptional regulation of circadian mRNA abundance rhythms based on the absence of a strong correlation between endogenous transcript abundance rhythms (RNA-seq) and the presumed transcriptional activity of 187 luciferase reporter genes [31]. However, the luciferase reporters contained complete gene-specific 5’ UTRs and 3’ UTRs (500 bp), which usually contain regulatory elements for posttranscriptional control [49-52] and were thus not suited to report promoter activity. The observed differences between mRNA and *luc* expression were likely due to growth conditions suppressing and supporting rhythmic conidiation, respectively.

In contrast to *Neurospora*, genome-wide analyses of circadian gene expression rhythms in mice and flies suggested a substantial contribution of posttranscriptional mechanisms [4,5,21]. Although the distribution of expression phases of ccgs was more diverse in mice and flies [4-7] than in *Neurospora*, a recent analysis suggested dawn and dusk as rush hours for circadian gene expression in various mouse tissues and organs [53].

**Figure 8:**
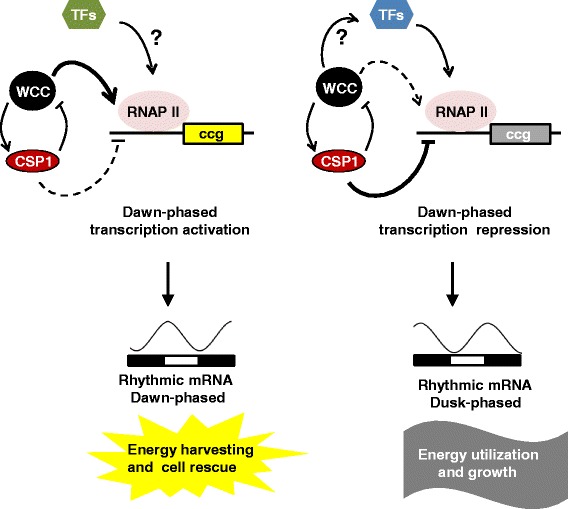
**Model of dawn- and dusk-phased transcription regulation.** Genes activated by the WCC are transcribed predominantly during subjective late night to early morning where the activity of WCC is high. The activity of other transcription factors (TFs) including CSP1 could modulate the exact phase and the amplitude of these dawn-phased genes. These dawn-specific genes are mainly involved in energy harvesting and cell rescue. The phases of CSP1 repressed genes are enriched around dusk. Yet unidentified TFs must be involved in rhythmic activation of these genes since the phase and amplitude, but not the rhythmic expression of tested genes, were affected in ∆*csp1.* Our data suggest that expression of these TFs could be under the control of WCC. Dusk-phased genes are involved in energy utilization and growth, indicating a temporal distinction of cellular pathways and functions coordinated by the circadian clock of *Neurospora.*

Expression of at least 80% of the genes that were rhythmically expressed in *wt* was directly or indirectly affected by the transcription activator WCC or the transcription repressor CSP1. Rhythmically expressed genes whose expression was reduced in the absence of WCC (n = 345) were predominantly morning specific. In contrast, rhythmically expressed genes downregulated by the morning-specific global repressor CSP1 (n = 298) were predominantly dusk-phased. Hence, WCC and CSP1 are major determinants of the expression phase of ccgs (Figure 8).

Surprisingly, the number of rhythmically expressed genes increased more than twofold in the absence of CSP1. This increase may reflect the interconnection between circadian gene expression and metabolism, since CSP1 expression is dependent on the glucose concentration in the medium [34]. The circadian transcriptomes of *wt* and Δ*csp1* were determined from cultures grown in high glucose medium, that is, under conditions when CSP1 is active. Under these conditions Δ*csp1*-specific ccgs might be repressed in *wt* and/or their rhythm might be blunted beyond the limit of detection. The rhythmic RNA abundances of the previously identified CSP1 target genes remained rhythmic in Δ*csp1* but showed phase shifts towards morning indicating that unknown TFs rhythmically activate their transcription.

The data strongly suggest that the complexity of the apparent circadian transcriptome is dependent on growth conditions. Recently, a similar phenomenon has been observed in mammals: feeding mice with a high-fat diet (HFD) resulted in phase advances or even loss of transcript rhythms, in addition to the appearance of new oscillating transcripts [54]. The appearance of new rhythmic transcripts was dependent on increased PPARγ activity. Integration of nutrient sensing TFs, such as CSP1 in *Neurospora* and *PPARγ* in mammals, into the core circadian gene network could modulate circadian output according to the needs of the cell under various nutrient conditions. The rich medium and constant conditions generally used to analyze circadian rhythms in *Neurospora* or mammalian cell culture may specifically disfavor and even suppress circadian gene expression rhythms, resulting in detection of a rather low number of ccgs.

An overruling distinction of dusk- and dawn-phased genes became evident when we analyzed the functional gene categories enriched in these two groups. Dawn-phased gene expression supports catabolic reactions whereas expression of dusk-phased genes supports anabolic reactions. This temporal separation of physiology could confer efficient utilization of available energy sources. Thus, during the day, when forward growth of *Neurospora* is fast but little biomass is produced, it may occupy and assess its environment and mobilize available resources (secretion of cellulases and degrading enzymes). During the day *Neurospora* seems to specifically protect itself from desiccation (synthesis of trehalose and glycerol), high temperature (heat shock proteins) and bacterial competitors (lysozyme). Towards the end of the day and during the night, when the environment becomes less hostile, genes required for protein synthesis (rRNA synthesis and ribosome assembly), DNA replication and cell division [55] (histones and DNA replication regulatory proteins) are upregulated to initiate cell growth and production of biomass in accordance with the previous assessment of available resources.

## Conclusions

Number, identity, phase and amplitude of rhythmically expressed genes, that is, the complexity of the circadian transcriptome, are likely a property of a complex metabolic network and depend on environmental conditions. The intimate link of circadian clocks with metabolism seems to be conserved in cyanobacteria, plants and animals [15,56-60]. Coordination of catabolic and anabolic function might thus be the major role of circadian clocks.

## Materials and methods

### *Neurospora* strains and culture conditions

*Neurospora* strains *wt* (FGSC#2489), ∆*csp1* (FGSC#11348) and ∆*wc2* (FGSC#11124) were acquired from Fungal Genetics Stock Center (FGSC, Manhattan, KS, USA). Vogel's medium (1 X) supplemented with 2% glucose, 0.5% L-arginine and 10 ng/ml biotin was used as a standard growth medium. For the circadian time course experiments, 200 ml medium in 500 ml flasks were inoculated with approximately 10^6^ conidia and grown in light for six hours for conidial germination. Afterwards cultures were entrained in 11 hour/11 hour light/dark cycles for 2 days and released to constant dark for 22 hours. Cultures were transferred in a staggered manner, so harvesting of the cultures was performed within 10 hours.

### Cellulase assay

Cultures were grown in Vogel’s medium with 2% Avicel as the carbon source for the cellulase assay. During harvesting, cultures were washed with three culture volumes of cold water to minimize extracellular cellulase contamination. Due to the adherence of *Neurospora* to Avicel we can measure extracellular cellulase that is trapped in the Avicel/*Neurospora* mesh in addition to intracellular cellulase in the secretory pathway. Extraction buffer (50 mM Tris–HCl pH 7.5, 150 mM NaCl, 1.5 mM MgCl_2_, 0.1% NP-40, 1 mM phenylmethylsulfonyl fluoride (PMSF), 1 μg/ml leupeptin, 1 μg/ml pepstatin A) was mixed with 500 μl ground mycelia and incubated on ice for approximately 30 minutes with frequent vortexing. The cell homogenate was centrifuged at 20,000 g at 4°C for 30 minutes. Protein concentration was determined by NanoDrop 1000 spectrometer. Cellulase activity was calculated by using Azo-CM-Cellulose (Megazyme, Bray, Ireland) according to the manufacturer's protocol with minor modifications. Briefly, 120 μg total protein extract diluted in 200 μl 100 mM NaOAc, pH 4.6 was mixed with 200 μl Azo-CM-Cellulose and incubated at 40°C for one hour with constant shaking. A 1 ml precipitation solution (300 mM NaOAc, 20 mM ZnAc, pH 5, 75% EtOH) was added to the protein-Azo-CM-Cellulose mixture and centrifuged at 10,000 g at room temperature for 10 minutes. The color of the supernatant was measured at 590 nm with a Jenway 6320D spectrophotometer. To generate the standard curve for cellulase activity (see Additional file 17: Figure S7C), cellulase from *Aspergillus niger* (1U/μg) (Sigma-Aldrich Chemie Gmbh Munich, Germany) was used.

### RNA analysis

RNA was extracted with peqGOLD TriFAST (peqLab, Erlangen, Germany) according to the manufacturer’s protocol. RNA was dissolved in 70 μl nuclease free water with 80u Ribolock RNAse inhibitor (ThermoScientific, Waltham, MA US). For the cDNA preparation Maxima First Strand cDNA Synthesis Kit (ThermoScientific, Waltham, MA US) was used. Transcript levels were analyzed by quantitative real-time PCR in 96-well plates with the StepOnePlus Real-Time PCR System (Life Technologies, NY, USA) using TaqMan Gene Expression Master Mix (Life Technologies, NY, USA). Primers and probes are listed in Additional file 18: Table S11. rRNA was used for normalization.

### Chromatin immunoprecipitation

ChIP was performed as described previously [32] by using specific antibody for serine-2 phosphorylated RNAPII C-terminal tail. Polyclonal anti-rabbit RNAPII Ser2-P was raised against the peptide (pS)PTSPSY(pS)PTSPS*C.* Primers and probes used for ChIP-PCRs are listed in Additional file 18: Table S11. *actin* gene (*ncu04173*) was used for normalization.

### RNA sequencing and ChIP sequencing

cDNA was prepared by using NEBNext® Ultra RNA Prep kit with NEBNext® Multiplex oligos according to the manufacturer’s instructions. ChIP DNA libraries were prepared with NEBNext® ChIP-Seq Library Prep Reagent Set for Illumina® with NEBNext® Multiplex oligos. A 2100 Bioanalyzer was used to check the size and the quality of the libraries. Un-paired sequencing with 50 bp reads was performed with a HiSeq 2000 at GeneCore EMBL Heidelberg for RNA-seq and by the BGI, Hong Kong, for ChIP-seq. Individual sequence reads for each run are available in the Sequence Read Archive (SRA) database under the study name PRJNA248256. Accession numbers for experiments and number of sequence reads are listed in Additional file 19: Table S12.

### High-throughput data analysis

Raw sequence reads were mapped to the *Neurospora crassa* genome (NC10) using Bowtie [61], where parameters were set to allow maximum three mismatches and suppress alignments which mapped to more than one location. Gene expression was quantified by the number of reads falling into the annotated exons. For analysis of the RNAPII-S2P ChIP-seq the reads that fall to the 500 bp window upstream of gene end position were counted. Normalization was carried out using the size factor formula as described [62].

In order to prevent a read contamination from neighboring genes, an ‘*Influence factor*’ was calculated as shown below:

$$ Influence\kern0.5em =\kern0.5em \frac{G/L{}_G}{\left(G\kern0.5em -\kern0.5em O\right)/\left(L{}_G\kern0.5em -\kern0.5em L{}_O\right)} $$

where *G* is the read count mapped to the gene, *L*_*G*_ is the length of the gene, *O* is the read count mapped to the overlapping region and *L*_*O*_ is the length of the overlap. The gene with an *Influence factor* larger than 2 was filtered from the RNA-seq and ChIP-seq analysis. The genes with low RNA levels (lower 20% of all annotated genes) were excluded from further analysis. In order to assign the cut-off for RNAPII-S2P ChIP-seq, a background read was calculated by counting the reads that fall into 500 bp windows from non-coding regions of the genome. Genes with a lower RNAPII-S2P signal compared to the median of the background reads were excluded from further analysis. Normalized RNA-seq and RNAPII-S2P reads are shown in Additional file 1: Table S1 and Additional file 13: Table S8 (∆*csp1* RNA-seq).

### Differential gene expression analysis

The read counts mapped to the genes were assumed to follow a negative binomial (NB) distribution:

$$ {G}_i\approx NB\left({\upmu}_i,{\upsigma}_i\right) $$

where μ_*i*_ is the mean and σ_*i*_ is the variance. σ_*i*_ was estimated based on the mean and variance correlation. A ‘*locfit*’ R package was used to fit the relationship between mean and variance. Adapting from the Robinson and Smyth Exact Test [63], the two-sided *P* value can be calculated by using the formula:

$$ p\kern0.5em =\kern0.5em \frac{\Sigma_{f\left(a,b\right)\le f\left({G}_{treat},\kern0.5em {G}_{control}\right)}f\left(a,b\right)}{\Sigma f\left(a,b\right)} $$

where $a+b\kern0.5em =\kern0.5em {G}_i^{treat}+{G}_i^{treat},a,b\kern0.5em \in \kern0.5em 0\dots \left({G}_i^{treat}+{G}_i^{treat}\right),{G}_i^{treat}$ is the reads count of *i*_*th*_ gene in treatment condition, ${G}_i^{treat}$ is the reads count of *i*_*th*_ gene in control condition, *f* (*a*, *b*) is the product of *f* (*a*) and *f* (*b*), which can be computed using ‘*dnbinom*’ of the R package.

### Identification of rhythmic RNA and RNAPII-S2P profiles

The ARSER [36] program was used to identify rhythmic RNA and RNAPII-S2P profiles. Period length was set between 18 to 26 hours. In order to detect rhythmic expression of genes that have very high expression levels in light (0 time point), we ran ARSER twice with and without the 0 time point. All genes that have ARSER detected RNA and/or RNAPII-S2P profiles were selected for further analysis. The ARSER output is shown in Additional file 3: Table S2. Independent phase and amplitude determination was performed by fitting the data to a sine wave using a negative binomial generalized linear model. The ‘*glm.nb*/function from MASS package of R was used and the period length was set as 22 hours.

### False discovery rate analysis

In total, 500 simulation data were generated for RNA and RNAPII-S2P by randomly shuffling the 12 time points by using the ‘sample’ function of R. The shuffled data were then analyzed by ARSER twice with and without 0 time point. It was observed that the occurrence of rhythmic genes follows an exponential decay function against the amplitude; exponential decay functions were fitted to both the original and the simulated data. The FDR was then computed by using the following formula:

$$ FDR(Amplitude)\kern0.5em =\kern0.5em \frac{f_{bg(Amplitude)}}{f_{ob(Amplitude)}\kern0.5em +\kern0.5em {f}_{bg(Amplitude)}}\times \kern0.5em  Weight(Amplitude) $$

Where, *f*_*bg*(*Amplitude*)_, *f*_*ob*(*Amplitude*)_ are the estimated occurrence of rhythmic genes against the amplitude using the fitted exponential decay function, and *Weight (Amplitude)* is introduced to remove the bias for the smaller number of occurrence when the amplitude is high. FDR according to Coverage is computed by using the same formula.

## Additional files

Additional file 1: Table S1. RNA-seq and RNAPII-S2P ChIPseq reads of *wt* time-course experiment. Additional file 2: Figure S1. Circadian RNA-seq and RNAPII-S2P ChIP-seq profiles of previously identified ccgs. (A) Time-resolved RNA-seq and ChIP-seq of RNAPII-S2P reads of the indicated genes were visualized with IGV genome browser [64]. Arrows indicate the transcription direction of genes. Bars and numbers on the right side of each image show the scale of the normalized reads. (B) Heat-maps of genes with a significant RNA abundance rhythm (*P* <0.05) and a non-significant RNAPII-S2P rhythm (*P* ≥0.05) in phase with the transcript rhythm. (C) Heat-maps of genes with a significant RNAPII-S2P occupancy rhythm (*P* <0.05) and a non-significant RNA abundance rhythm (*P* ≥0.05) in phase with the transcribing RNAPII. Note that the expression patterns of the significant and their corresponding non-significant rhythms are similar. Additional file 3: Table S2. ARSER output of time-course experiments with and without ‘0’ time point. Additional file 4: Table S3. Analysis of rhythmic genes according to significance and amplitude. Additional file 5: Table S4. Rhythmically transcribed genes without in phase transcript abundance rhythms. Additional file 6: Table S5. Genes showing significant RNA abundance rhythms without rhythmic RNAPII-S2P occupancy profiles. Additional file 7: Figure S2. Comparison of rhythmically expressed genes identified by Hurley *et al*. and in this study. (A) Venn diagrams showing the overlap between rhythmic genes identified by Hurley *et al*. by RNA-seq and this study by RNA-seq and RNAPII ChIP-seq. (B) Box-plots showing the RNA coverage of genes identified by both studies (grey) and only by Hurley *et al*. (light-red). (C-D) Box-plots showing amplitudes of RNA oscillations of the genes shown in B. (C) Amplitudes (peak/trough) of sine waves fitted to our RNA-seq data. (D) Amplitudes (peak - trough in RPKM, as defined by Hurley *et al*.) of the three RNA-seq replicates of Hurley *et al*. (E) Heat-maps showing RNA and RNAPII-S2P profiles of 327 genes that are identified by both studies. (F-G) Examples of rhythmically transcribed genes (F) RNAPII-S2P ChIP-Seq profiles of the indicated genes. Sequence reads were double plotted. Light- and dark-shaded areas correspond to subjective day (CT 0 to 12) and night (CT 12 to 0), respectively. The expression levels (Y-axis) are shown as mapped normalized reads. (G) Two independent RNAPII-S2P ChIP-PCR analyses showing oscillation of the abundance rhythms of transcribing RNAPII at indicated genes. Note that Hurley *et al*. assigned *ccg1* and *ccg9* as non-rhythmic based on luciferase reporters. RNAPII-S2P CHIP-PCRs of two other rhythmically transcribed genes (*ncu07318* and *frq*) are shown. Additional file 8: Figure S6. Cell cycle genes encoding homologs or subunits of complexes are co-expressed. Double plotted reads of the indicated genes are shown. (A) *mcm* homologues (encoding for the subunits of DNA replication licensing factor), (B) *rpa* homologues (encoding for the subunits of hetero-trimeric Replication Protein A), (C) *rfc* homologues (encoding for the subunits of the hetero-pentameric clamp loader complex), and (D) core histones. Only the genes labeled by an asterisk were detected with a significant RNA abundance and/or RNAPII-S2P occupancy rhythm. Additional file 9: Table S10. FunCat analysis of dawn- and dusk-phased genes. Additional file 10: Table S6. RNA-seq of light-grown *wt* and ∆*wc2* strains. Additional file 11: Table S7. Comparison of CSP1 regulated genes with rhythmic genes. Additional file 12: Figure S3. Phases of rhythmic genes indirectly controlled by WCC and CSP1*.* (A) Bar plots of phase distribution of rhythmic genes that are upregulated in (A) ∆*wc2* or (B) *csp1*_*OE*_ *.* Numbers of dawn- and dusk-phased genes are indicated in red and blue, respectively. The dotted lines correspond to the phase distribution expected on the basis of 1,407 rhythmic genes. (C and D) Venn diagrams showing the comparison of genes differentially regulated in ∆*wc2* and *csp1*_*OE*_. Additional file 13: Table S8. RNA-seq time-course analysis of ∆*csp1* strain. Additional file 14: Figure S4. CSP1 suppresses rhythmic expression of a large number of genes. (A) Heat-map of genes with similar expression phase in *wt* and ∆*csp1.* (B) Heat-map of genes that are rhythmically expressed in ∆*csp1* but not in *wt*. (C) Double plot of circadian expression profiles of selected genes in *wt* and ∆*csp1. frq* is included as a control. Additional file 15: Table S9. Genes showing significant RNA abundance rhythms in ∆*csp1* but not in *wt*. Additional file 16: Figure S5. The biochemical pathways for (A) arabinose/xylose utilization and (B) trehalose synthesis. All genes encoding for the indicated enzymes have rhythmic RNA and/or RNAPII-S2P profiles. The genes, except *xry-1,* are expressed with a morning-specific phase. Additional file 17: Figure S7. Ccgs upregulated when *Neurospora* is grown on plant tissue or cellulose are predominantly morning-specific. (A) Circadian expression profiles of the overlapping group of genes that are rhythmically expressed (1,407 genes identified in this study) and upregulated when *Neurospora* is grown in liquid medium with plant tissue or cellulose (304 genes identified by Tian *et al*. [48]). Rhythmic and upregulated in medium containing miscanthus stems (left panel), Avicel (middle panel), and Avicel and miscanthus stems (right panel), respectively. NCU numbers of the genes are shown on the right side of the heat-maps. The number of overlapping genes is indicated at the bottom of the heat-maps. (B) Standard curve used to calculate cellulase activity of *wt* and ∆*csp1* strains in Figure 7. Additional file 18: Table S11. Primers and probes used in this study. Additional file 19: Table S12. Accession numbers of sequencing reads in SRA database.
