# Supplementary figures and images for: Dawn- and dusk-phased circadian transcription rhythms coordinate anabolic and catabolic functions in Neurospora
Source: BMC Biol. 2015 Feb 24;13:17. doi: 10.1186/s12915-015-0126-4 (PMC4381671; doi:10.1186/s12915-015-0126-4)

Supplemental Figure 1

A

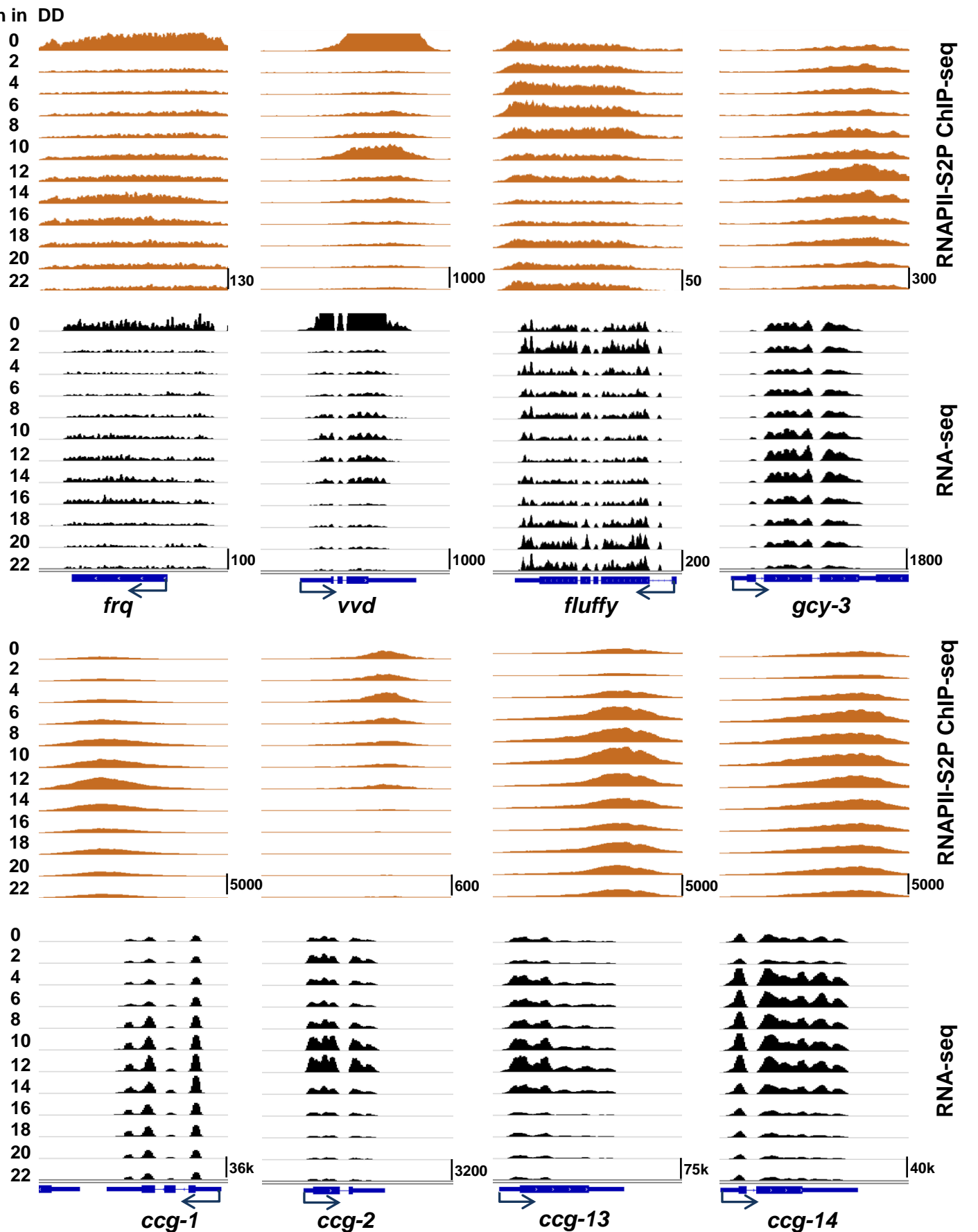

Supplemental Figure 1

B

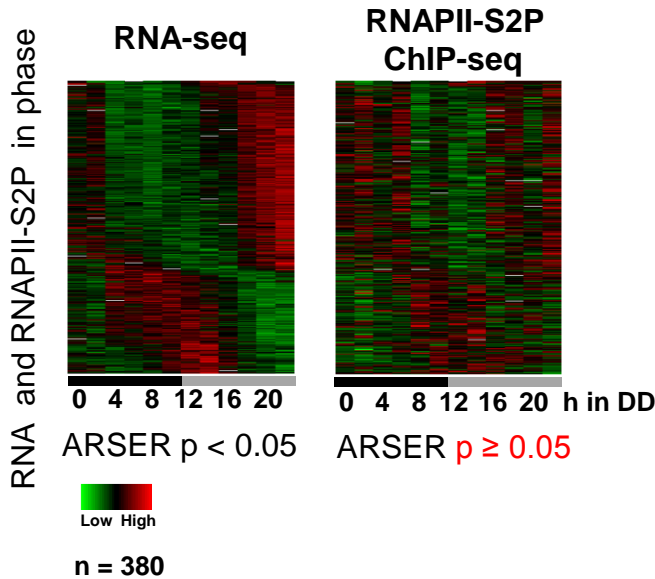

C

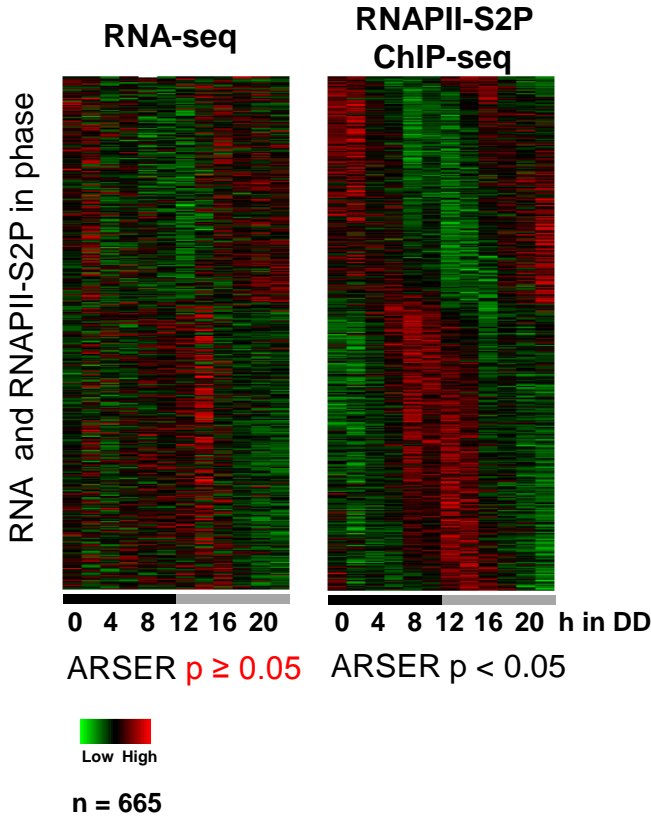

Supplement: Additional file 2: Figure S1. — Circadian RNA-seq and RNAPII-S2P ChIP-seq profiles of previously identified ccgs. (A) Time-resolved RNA-seq and ChIP-seq of RNAPII-S2P reads of the indicated genes were visualized with IGV genome browser [64]. Arrows indicate the transcription direction of genes. Bars and numbers on the right side of each image show the scale of the normalized reads. (B) Heat-maps of genes with a significant RNA abundance rhythm (P <0.05) and a non-significant RNAPII-S2P rhythm (P ≥0.05) in phase with the transcript rhythm. (C) Heat-maps of genes with a significant RNAPII-S2P occupancy rhythm (P <0.05) and a non-significant RNA abundance rhythm (P ≥0.05) in phase with the transcribing RNAPII. Note that the expression patterns of the significant and their corresponding non-significant rhythms are similar. [file 12915_2015_126_MOESM2_ESM.pdf]

Supplemental Figure 2

A

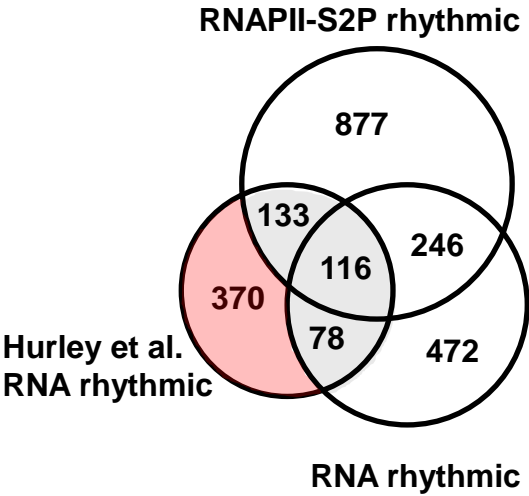

B

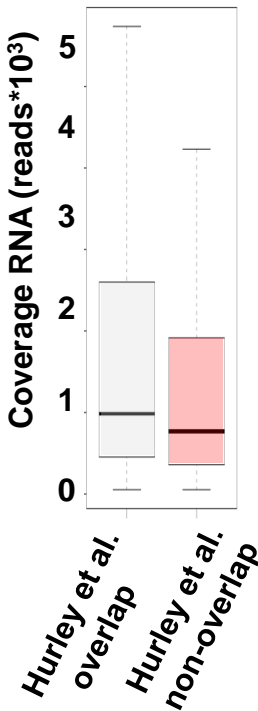

C

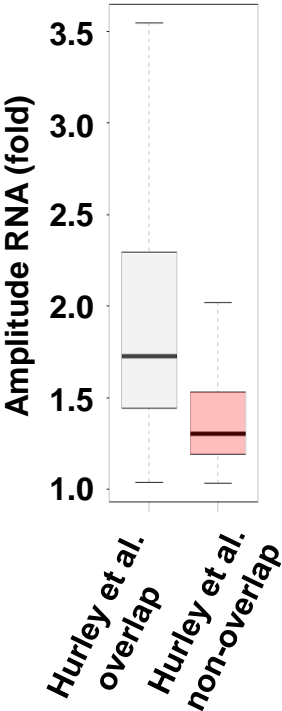

D

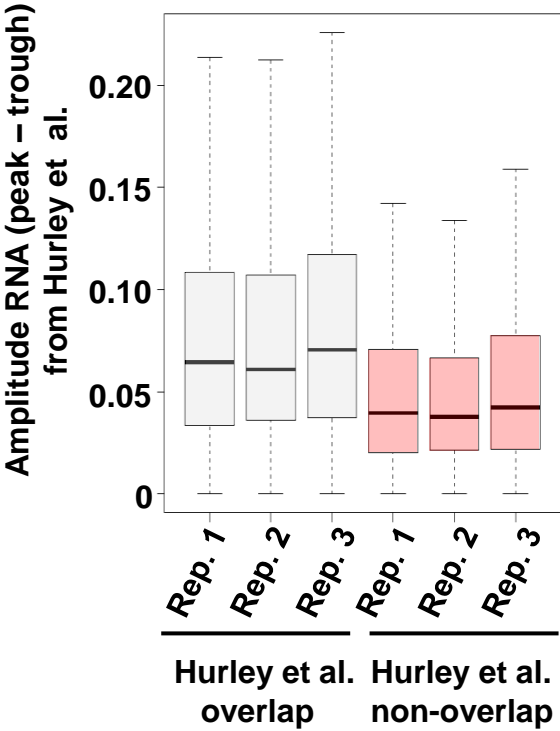

E

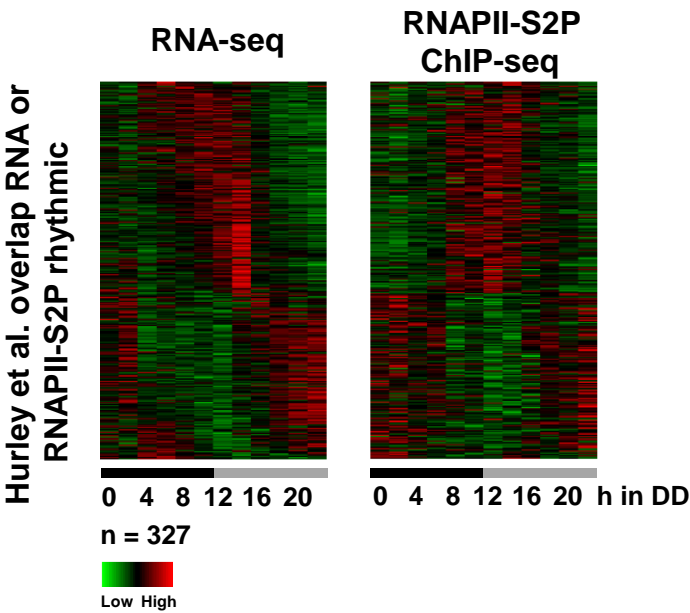

F

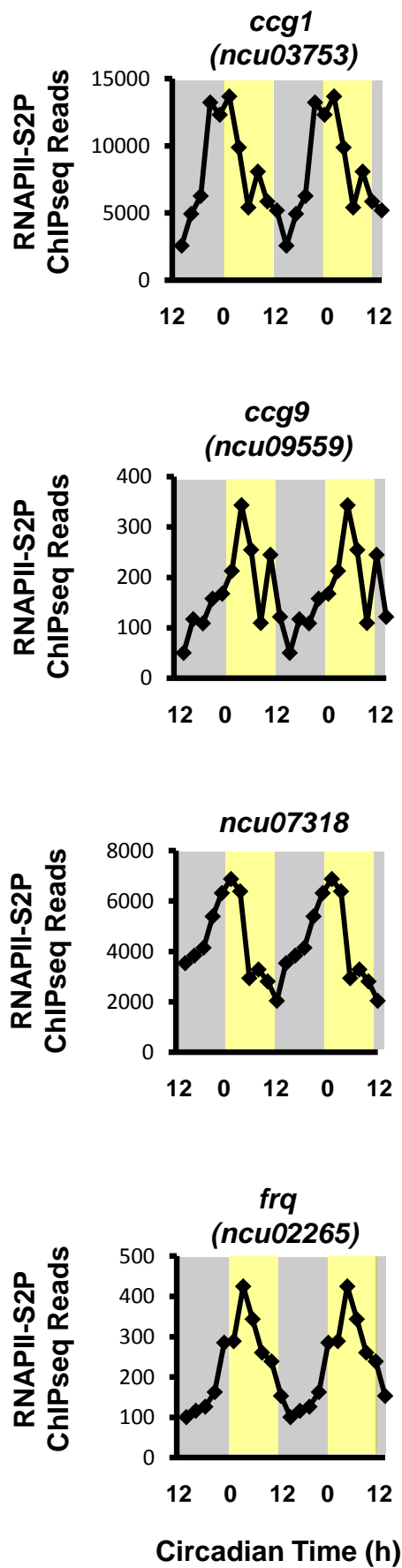

G

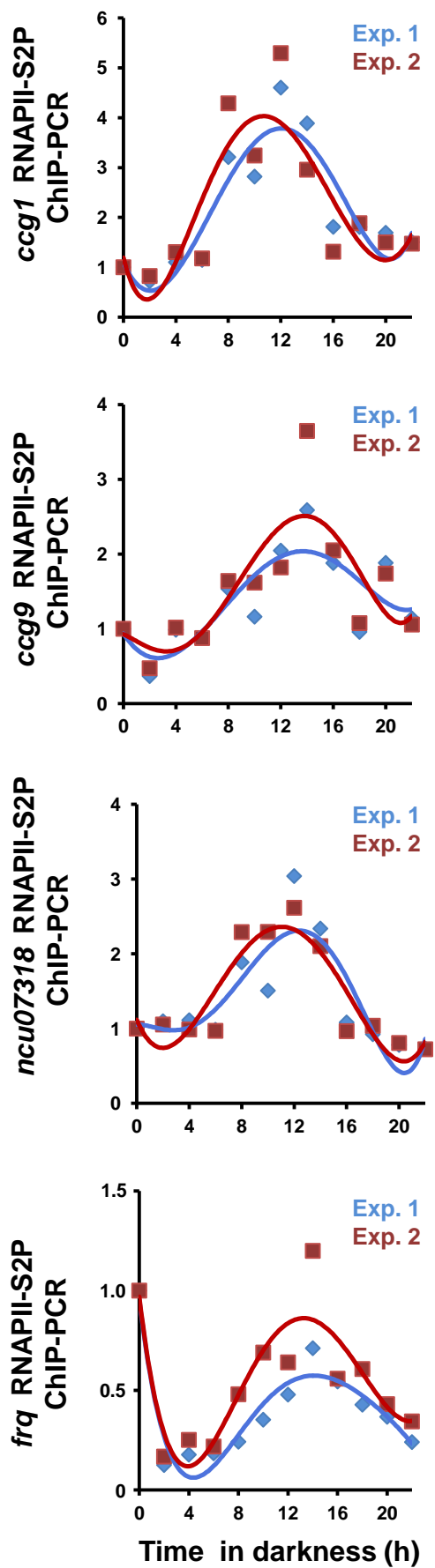

Supplement: Additional file 7: Figure S2. — Comparison of rhythmically expressed genes identified by Hurley et al. and in this study. (A) Venn diagrams showing the overlap between rhythmic genes identified by Hurley et al. by RNA-seq and this study by RNA-seq and RNAPII ChIP-seq. (B) Box-plots showing the RNA coverage of genes identified by both studies (grey) and only by Hurley et al. (light-red). (C-D) Box-plots showing amplitudes of RNA oscillations of the genes shown in B. (C) Amplitudes (peak/trough) of sine waves fitted to our RNA-seq data. (D) Amplitudes (peak - trough in RPKM, as defined by Hurley et al.) of the three RNA-seq replicates of Hurley et al. (E) Heat-maps showing RNA and RNAPII-S2P profiles of 327 genes that are identified by both studies. (F-G) Examples of rhythmically transcribed genes (F) RNAPII-S2P ChIP-Seq profiles of the indicated genes. Sequence reads were double plotted. Light- and dark-shaded areas correspond to subjective day (CT 0 to 12) and night (CT 12 to 0), respectively. The expression levels (Y-axis) are shown as mapped normalized reads. (G) Two independent RNAPII-S2P ChIP-PCR analyses showing oscillation of the abundance rhythms of transcribing RNAPII at indicated genes. Note that Hurley et al. assigned ccg1 and ccg9 as non-rhythmic based on luciferase reporters. RNAPII-S2P CHIP-PCRs of two other rhythmically transcribed genes (ncu07318 and frq) are shown. [file 12915_2015_126_MOESM7_ESM.pdf]

Supplemental Figure 6

A

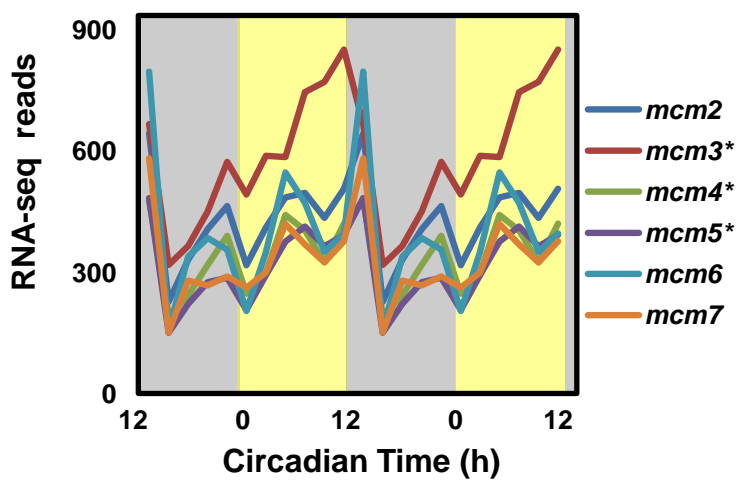

B

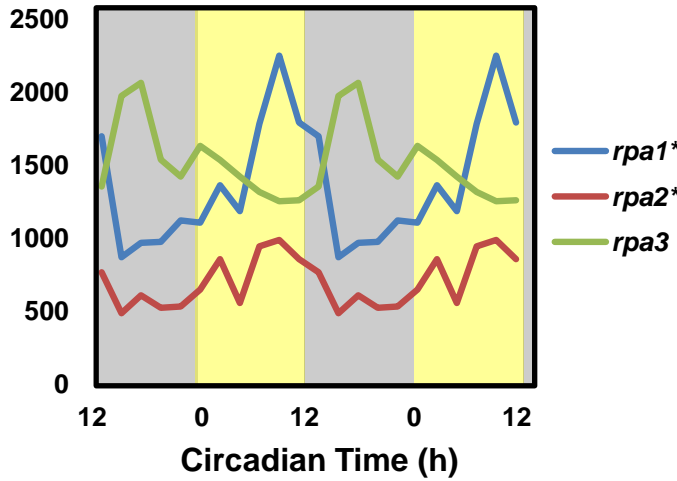

C

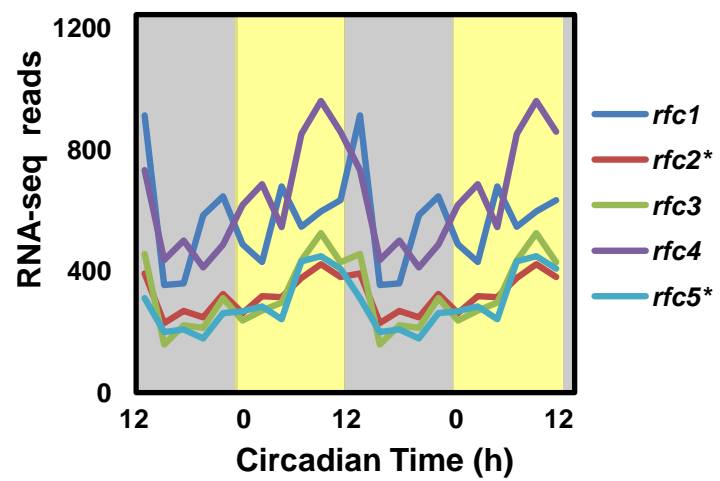

D

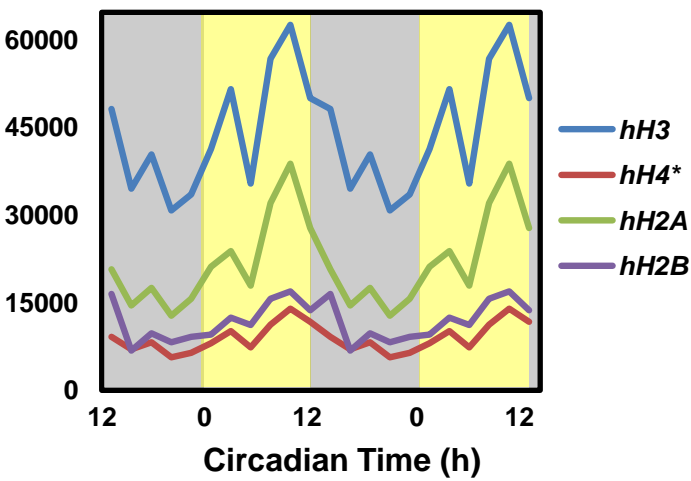

Supplement: Additional file 8: Figure S6. — Cell cycle genes encoding homologs or subunits of complexes are co-expressed. Double plotted reads of the indicated genes are shown. (A) mcm homologues (encoding for the subunits of DNA replication licensing factor), (B) rpa homologues (encoding for the subunits of hetero-trimeric Replication Protein A), (C) rfc homologues (encoding for the subunits of the hetero-pentameric clamp loader complex), and (D) core histones. Only the genes labeled by an asterisk were detected with a significant RNA abundance and/or RNAPII-S2P occupancy rhythm. [file 12915_2015_126_MOESM8_ESM.pdf]

Supplemental Figure 3

A

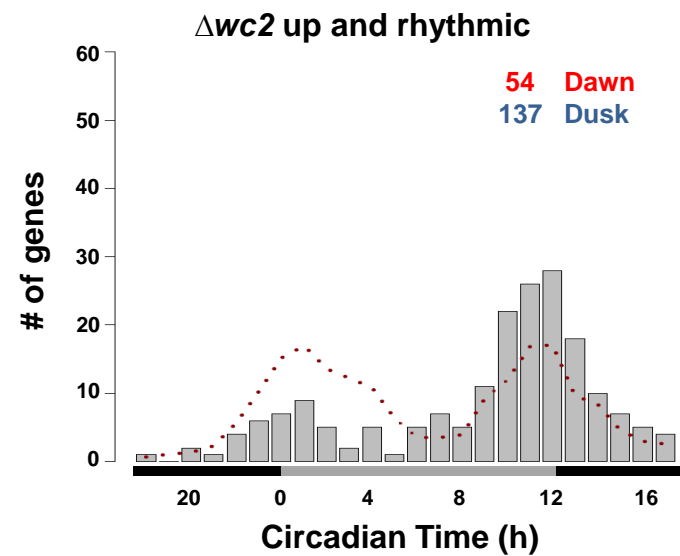

B

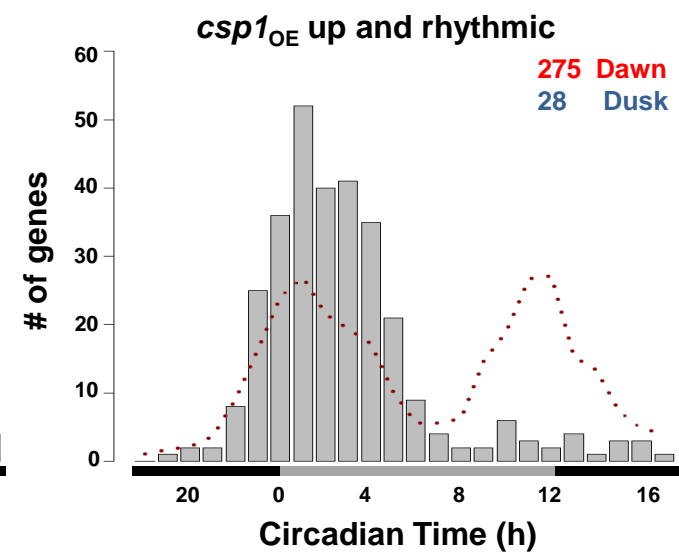

C

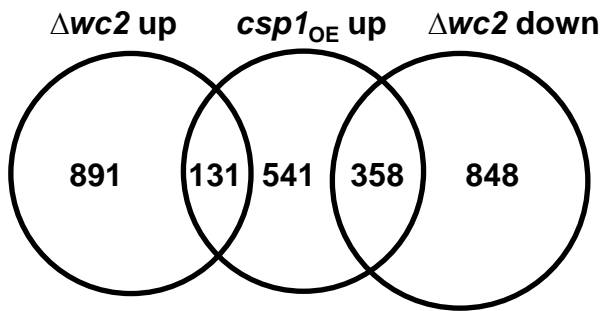

D

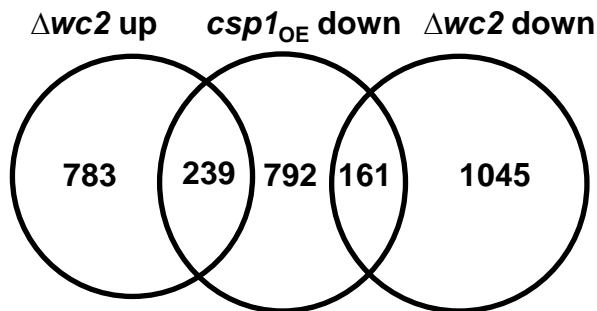

Supplement: Additional file 12: Figure S3. — Phases of rhythmic genes indirectly controlled by WCC and CSP1. (A) Bar plots of phase distribution of rhythmic genes that are upregulated in (A) ∆wc2 or (B) csp1 OE . Numbers of dawn- and dusk-phased genes are indicated in red and blue, respectively. The dotted lines correspond to the phase distribution expected on the basis of 1,407 rhythmic genes. (C and D) Venn diagrams showing the comparison of genes differentially regulated in ∆wc2 and csp1 OE. [file 12915_2015_126_MOESM12_ESM.pdf]

Supplemental Figure 4

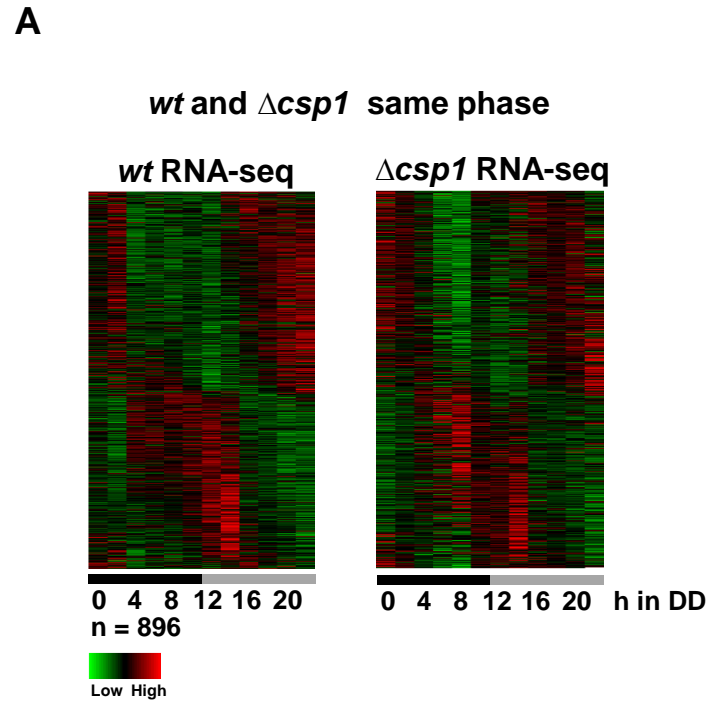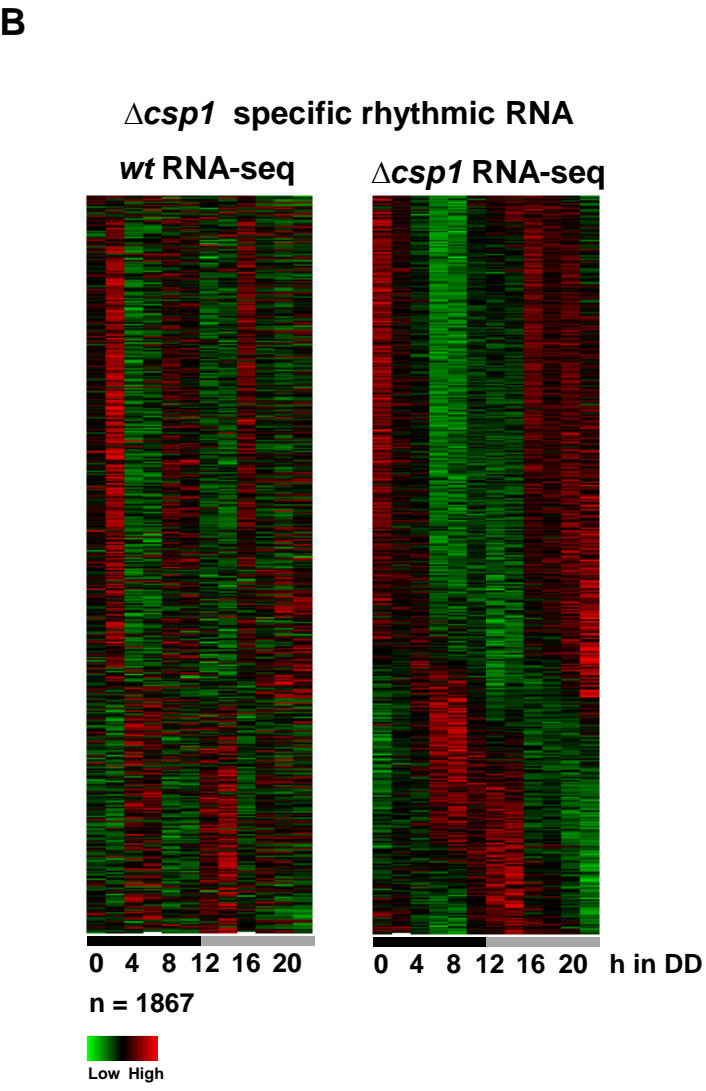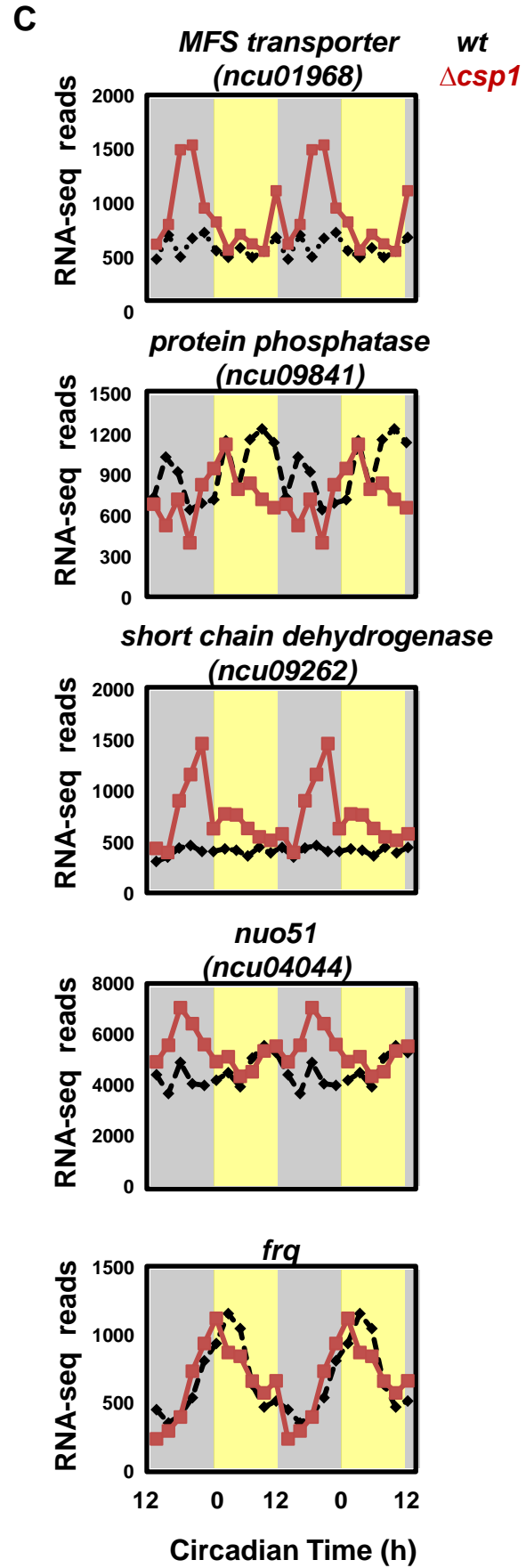

Supplement: Additional file 14: Figure S4. — CSP1 suppresses rhythmic expression of a large number of genes. (A) Heat-map of genes with similar expression phase in wt and ∆csp1. (B) Heat-map of genes that are rhythmically expressed in ∆csp1 but not in wt. (C) Double plot of circadian expression profiles of selected genes in wt and ∆csp1. frq is included as a control. [file 12915_2015_126_MOESM14_ESM.pdf]

Supplemental Figure 5

A

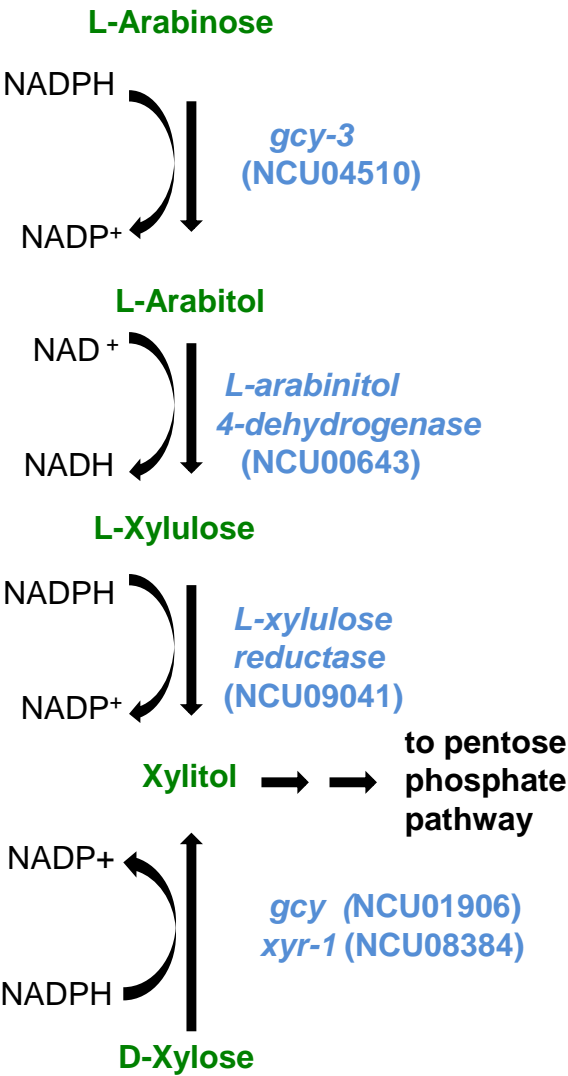

B

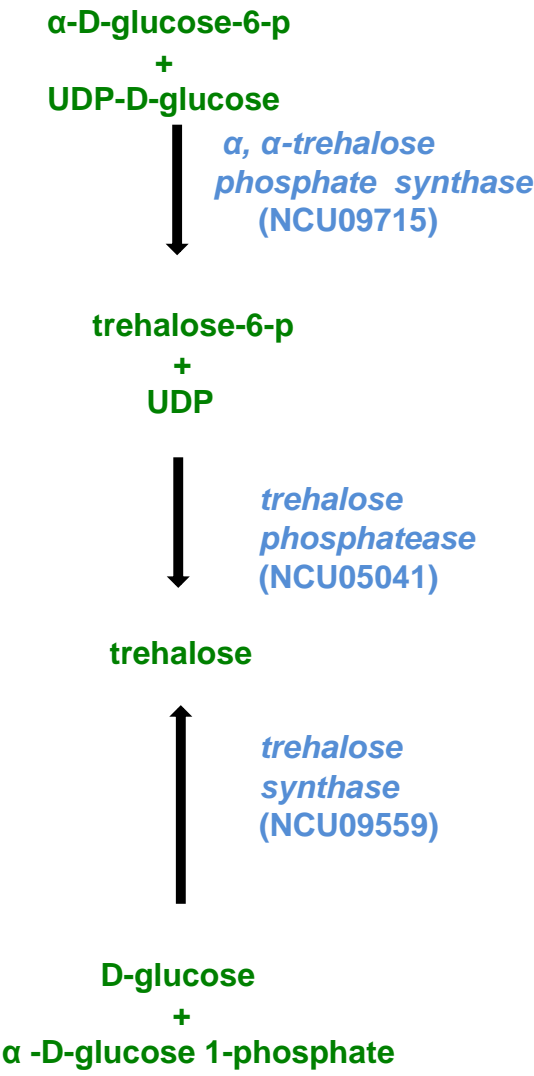

Supplement: Additional file 16: Figure S5. — The biochemical pathways for (A) arabinose/xylose utilization and (B) trehalose synthesis. All genes encoding for the indicated enzymes have rhythmic RNA and/or RNAPII-S2P profiles. The genes, except xry-1, are expressed with a morning-specific phase. [file 12915_2015_126_MOESM16_ESM.pdf]

Supplemental Figure 7

A

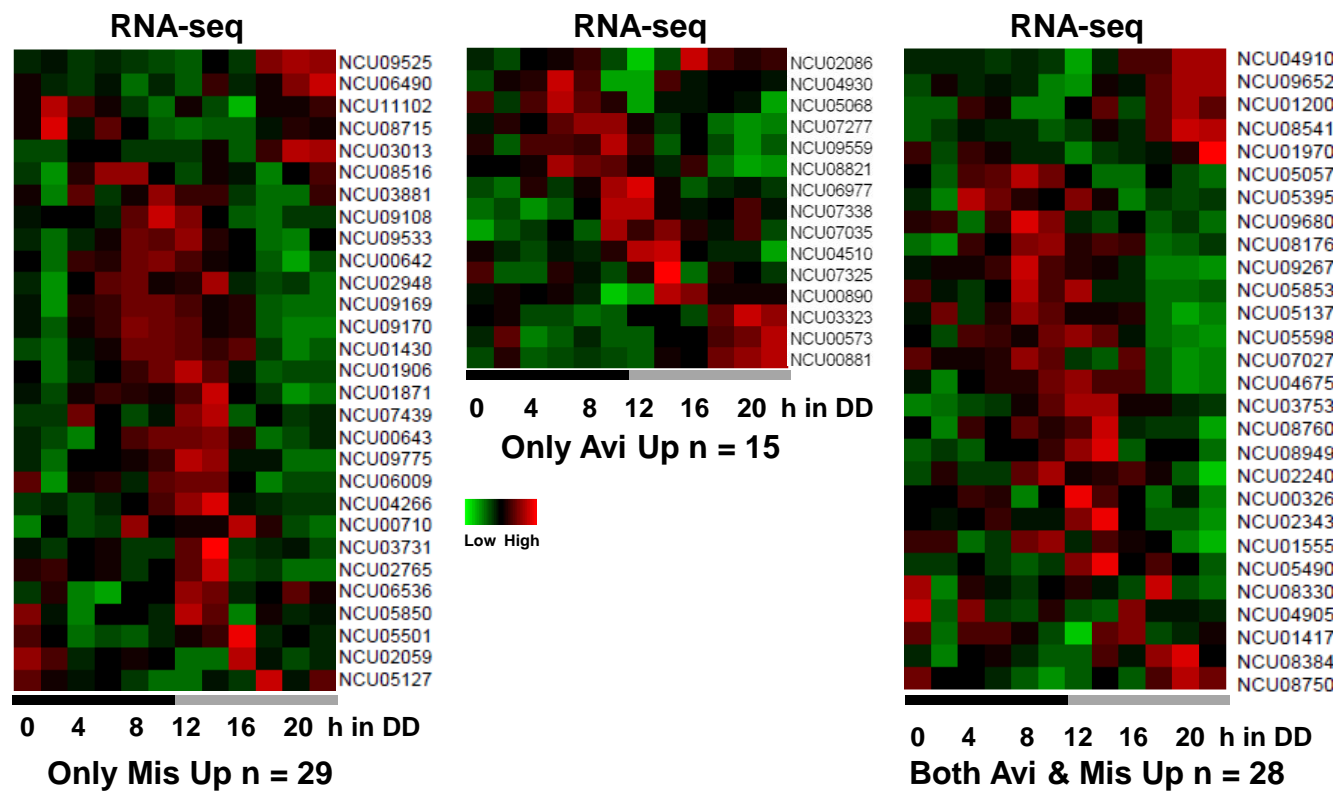

B

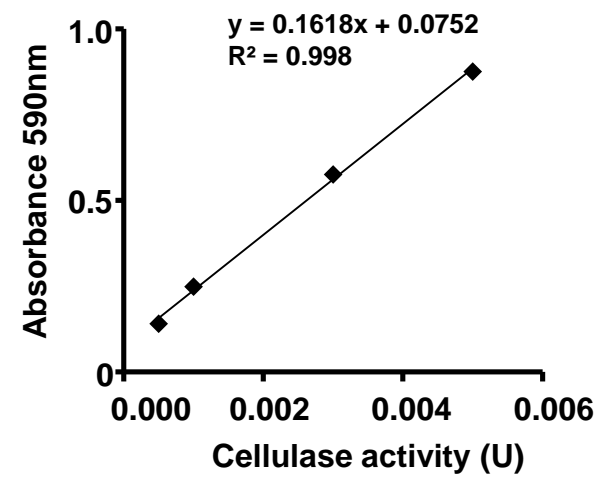

Supplement: Additional file 17: Figure S7. — Ccgs upregulated when Neurospora is grown on plant tissue or cellulose are predominantly morning-specific. (A) Circadian expression profiles of the overlapping group of genes that are rhythmically expressed (1,407 genes identified in this study) and upregulated when Neurospora is grown in liquid medium with plant tissue or cellulose (304 genes identified by Tian et al. [48]). Rhythmic and upregulated in medium containing miscanthus stems (left panel), Avicel (middle panel), and Avicel and miscanthus stems (right panel), respectively. NCU numbers of the genes are shown on the right side of the heat-maps. The number of overlapping genes is indicated at the bottom of the heat-maps. (B) Standard curve used to calculate cellulase activity of wt and ∆csp1 strains in Figure 7. [file 12915_2015_126_MOESM17_ESM.pdf]
